# Market survey predictions on the future of US Pap testing

**DOI:** 10.4103/1742-6413.55885

**Published:** 2009-09-18

**Authors:** R Marshall Austin, Barbara Benstein, Joel Bentz, Sandra Bigner, Gregory G Freund, Gregory La Rocco, Ibrahim Ramzy, Lynnette Savaloja, Vinod B Shidham

**Affiliations:** Address: Department of Pathology, Magee-Women's Hospital of University of Pittsburgh Medical Center, 300 Halket Street, Pittsburgh, PA 15213, USA

Predicting the future in almost any realm of human endeavor is a challenging and humbling undertaking, given the large number of variables often at play, uncertainty as to how known variables impact each other, the frequent presence of significant but largely unrecognized variables, and the even more daunting task of estimating timing in the unfolding of future changes. With the identification of human papilloma virus (HPV) as the dominant etiologic agent of cervical cancer,[1,2] the introduction of FDA-approved adjunctive HPV DNA testing with cytology as early forms of etiology-based screening[3,4] and the recent introduction of FDA-approved vaccination for the two leading high-carcinogenic-risk HPV types,[5] it is perhaps not surprising that recent predictions about the future structure of cervical cancer prevention efforts in the USA and the size and scope of the role of the Papanicolaou test in US cervical cancer screening have varied greatly.[6–13]

In an effort to help members assess the future of the Pap test market in the USA, the American Society of Cytopathology (ASC) recently contracted with an external research group FGI Research of Chapel Hill, North Carolina, to survey a diverse group of US Pap test providers on their personal expectations for future Pap testing volume in their practices. Questions to be asked of Pap test providers were initially developed by the ASC Long Range Planning Committee and subsequently edited by the ASC Executive Board and FGI Research. Along with the specific questions asked, the Survey findings are presented here for further consideration by the ASC membership [see PDF of FGI PowerPoint presentation - Report of findings - http://www.cytojournal.com/articles/2009/6/1/images/CytoJournal_2009_6_1_17_55885_sm1.pdf].

## COMMENTARY

The respondents' predictions that 5 years from now providers expect to order roughly the same number of Pap tests that they order now per 30-day period can be interpreted in several ways. People often expect present circumstances to continue indefinitely, even as prior experience tells them that such expectations may often prove unrealistic.

Nevertheless, we believe that this major prediction of the market survey underscores the inherent conservatism and resistance to change in the current US cervical cancer prevention system, one grounded in multigenerational expectations of US women that they should present themselves for periodic (opportunistic) Pap-based wellness visits and prevention services to (predominantly private practice) primary care providers.[14,15]

Even gradual and minor changes to this opportunistic US screening system could present major challenges for overall screening compliance and effectiveness in the absence of a programmatic organized system such as the systems present in much of Europe and Australia.[16,17] Some suspect that this market-based conservatism substantially accounts for the still limited US adoption of routine Pap and HPV co-testing in women 30 and older compared to the more widespread adoption for reflex HPV testing after ASCUS Pap results.[8] The even more limited adoption of recommended extended screening intervals after “double-negative” Pap and HPV co-test results[18–20] further reflects market resistance to changes perceived as interfering with traditional annual women's wellness visits.[14] Intrinsic market conservatism is also consistent with the observation that that three-quarters of providers surveyed believe that the trends they are anticipating for the next 5 years will continue into the next 10–15 years.

Despite prevailing expectations by many providers that little will change, the survey does indicate that providers recognize several new factors which have already had an impact on numbers of Pap tests they have ordered in the recent past and which they also realize could further impact the number of Pap tests they will order in the future. Clinical guidelines on the recommended frequency of screening[18–20] in particular are acknowledged (blamed) by gynecologists as having negatively influenced the number of Pap tests ordered in the recent past, a trend they expect to continue. Despite the perceived influence of guidelines on Pap volume, respondents also commonly reported that they often simply elect not to follow guidelines. This tendency has been reported in a variety of clinical settings.[21–24] Adjunctive HPV DNA testing is also identified as a factor decreasing Pap testing, both with reflex HPV testing after ASCUS (presumably due to fewer repeat Paps) and with Pap and HPV co-testing in women 30 and older (increased recommended screening intervals). Insurance coverage is also acknowledged as a significant factor potentially causing fewer Pap tests.[8] Few respondents expect the HPV vaccine to eliminate the need for Pap testing in the foreseeable future, and until population-based data on the performance of cytology, HPV testing, and alternative screening or triage interventions become available, modifying current screening guidelines have been recently acknowledged as premature.[25]

One confusing finding in the survey is the expectation by 43% of respondents that they “foresee HPV DNA testing to be used for primary screening with cytology as triage.”

Among the respondents reporting this expectation, most said that they expect this change in as little as 2–5 years. Given the potential of primary HPV testing with cytology triage (cytology only after a positive HPV test) to substantially decrease Pap testing,[26,27] this finding is inconsistent with other survey data, and therefore we believe that respondents did not accurately understand this question. Since HPV DNA testing is already used for “primary screening” in the form of FDA-approved Pap and HPV co-testing, we believe that it is likely that many respondents simply misunderstood “cytology triage” as Pap and HPV co-testing. The reported answers to the survey question are then perhaps best interpreted as reflecting expectations that the option of Pap and HPV co-testing, now reportedly selected for testing by around 25–30% of women 30 and older, will experience continued, gradual increased market uptake.

Many factors could significantly impact the likelihood of realizing the survey respondents' expectations for gradual and limited change in Pap testing. A severe economic downturn, for example, has already increased pressure for national healthcare reform and cost-containment measures.[28,29] These pressures could play into the hands of those calling for increased screening intervals and restricted guideline-based reimbursement.[8] On the other hand, any move in this direction is likely to be opposed by women consumers who believe that recommendations to screen later and less frequently stem from financial considerations rather than balanced safety concerns.[30] If an HPV DNA test were at some point in the next decade to become FDA approved for primary screening with cytology triage, as proposed by some in Europe,[26] more significant changes in screening practices could certainly ensue. For now, these experiments appear to more likely to unfold in national screening programs in Europe and elsewhere overseas. For the foreseeable future, cervical cancer screening will remain crucial to cervical cancer prevention. Vaccination at this point is for cervical cancer prevention in the next generation of US women. FDA-approved cytology-based screening remains in the USA the most effective prevention modality for the current generation of women.

## COMPETING INTEREST STATEMENT BY ALL AUTHORS

No competing interest to be declared by any of the authors.

## AUTHORSHIP STATEMENT BY ALL AUTHORS:

All authors declare that we qualify for authorship as defined by ICMJE http://www.icmje.org/#author.
